# Deep-Learning-Based Assessment of Breast Arterial Calcification on Mammography: Technical Validation, Cardiovascular Prognostic Value, and Clinical Translation—A Narrative Review

**DOI:** 10.3390/medsci14060672

**Published:** 2026-10-14

**Authors:** Alexandru Carauleanu, Amelian M. Bobu, Elena Porumb-Andrese, Emilia Patrascanu, Camelia Tamas, Andrei Ionut Cucu, Roxana Covali, Iulian Prutianu, Iustina Solomon-Condriuc, Vlad Porumb, Catalin M. Buzduga

**Affiliations:** 1 Faculty of Medicine, Grigore T. Popa University of Medicine and Pharmacy Iasi, 700115 Iasi, Romania; ale.carauleanu@umfiasi.ro (A.C.); elena.andrese1@umfiasi.ro (E.P.-A.); patrascanu.emilia@umfiasi.ro (E.P.); ana.covali@umfiasi.ro (R.C.); pruty04@gmail.com (I.P.); iustina_condriuc@yahoo.com (I.S.-C.); vlad.porumb@umfiasi.ro (V.P.); catalin.buzduga@umfiasi.ro (C.M.B.); 2 Faculty of Medicine and Biological Sciences, Stefan cel Mare University of Suceava, 720229 Suceava, Romania; andrei.cucu@usm.ro

**Keywords:** breast arterial calcification, mammography, artificial intelligence, cardiovascular risk, cardiovascular disease, women’s cardiovascular health

## Abstract

Breast arterial calcification (BAC) is a manifestation of medial arterial calcification frequently detected incidentally on screening mammography. Increasing evidence suggests that BAC may also serve as a marker of cardiovascular risk. This narrative review critically synthesizes the evidence on artificial intelligence (AI)-based detection, segmentation, and quantification of BAC. It also evaluates the cardiovascular prognostic significance of automatically quantified BAC and examines the requirements for clinical translation. A targeted literature search of PubMed/MEDLINE, Scopus, Web of Science, and Google Scholar was conducted for English-language publications available from database inception through 3 September 2026, with additional studies identified from reference lists. Deep-learning approaches included convolutional neural networks, U-Net-derived segmentation architectures, transfer-learning models, and semi-supervised methods. Reported technical performance varied according to task and validation strategy. Representative segmentation models achieved Dice/F1 scores ranging from 0.666 to 0.756 and Jaccard indices from 0.581 to 0.614. Case-level BAC detection achieved areas under the receiver operating characteristic curve (AUCs) of up to 0.95–0.96 in selected test cohorts. However, external validation, learning-curve reporting, multivendor evaluation, and robustness assessment remained inconsistent. Prognostic studies demonstrated graded associations between automated BAC burden and cardiovascular outcomes. In a cohort of 21,514 women without known cardiovascular disease, age-adjusted BAC burden was associated with major adverse cardiovascular events (MACEs) among women classified as low risk, with adjusted hazard ratios (HRs) of 1.66 and 2.31 for BAC below and above the age-specific median, respectively. In 123,762 women from two healthcare systems, MACE risk increased progressively with BAC burden after adjustment using the Predicting Risk of Cardiovascular Disease EVENTs (PREVENT) equations, reaching approximately 2.8- to 3.3-fold higher risk with severe BAC. Nevertheless, incremental predictive improvement may be small—in one study, the addition of BAC changed the 5-year AUC from 0.645 to 0.646. Overall, available evidence supports the feasibility of scalable AI-based BAC assessment and demonstrates consistent associations between AI-quantified BAC and cardiovascular outcomes. However, technical performance and independent prognostic associations alone do not establish clinical utility. Standardized BAC definitions, genuinely independent external validation, and prospective implementation studies are required before BAC-guided cardiovascular management can be recommended.

## 1. Introduction

Despite substantial advances in prevention and treatment, cardiovascular disease (CVD) remains the leading cause of death among women worldwide, exceeding the mortality attributable to all forms of cancer combined and contributing substantially to disability—particularly after menopause, when hormonal changes interact with aging and accumulating cardiometabolic risk factors [1,2,3]. Ischemic heart disease accounts for approximately 47% of cardiovascular deaths among women, making it the largest contributor to female cardiovascular mortality worldwide [1]. Cardiovascular health, however, is not affected uniformly across sexes: women and men differ in the distribution of cardiovascular risk factors, mechanisms of disease, symptom profiles, clinical trajectories, and responses to therapeutic interventions [4]. These differences result from the interplay between biological factors—including hormonal, chromosomal, and reproductive characteristics—and gender-related determinants such as lifestyle, psychosocial stress, and access to healthcare [5].

Historically, evidence informing cardiovascular prevention and treatment was derived predominantly from male populations [6]. Although the representation of women in cardiovascular research and the scientific focus on female cardiovascular health have improved, meaningful gaps remain in risk recognition, timely diagnosis, access to appropriate treatment, and clinical outcomes. These persistent inequalities led the 2024 Lancet Regional Health–Europe Commission to identify women among the populations that continue to experience disproportionate cardiovascular health disparities [7]. This reinforces the need for prevention strategies that more accurately capture cardiovascular risk in women, including individuals who may be classified as being at low or intermediate risk by conventional prediction models [8].

One approach is to derive cardiovascular information from imaging examinations already performed for other clinical indications. Mammography, routinely used for breast cancer screening, may also reveal breast arterial calcification (BAC)—a vascular finding traditionally considered incidental [8]. Accumulating evidence has associated BAC with cardiovascular risk factors, systemic vascular disease, and subsequent cardiovascular events [9,10]. Therefore, BAC assessment may extend the clinical value of mammography beyond cancer detection by enabling opportunistic cardiovascular risk evaluation without an additional imaging procedure or radiation exposure [9].

BAC is identified in approximately 10–13% of women undergoing screening mammography, although its prevalence varies considerably according to age, cardiometabolic profile, and the method used for detection [11]. Unlike coronary artery calcification, which primarily reflects calcium deposition within atherosclerotic plaques in the arterial intima, BAC predominantly represents medial arterial calcification [12,13]. This process is characterized by calcium deposition within the tunica media, phenotypic transformation of vascular smooth muscle cells, and progressive arterial stiffening rather than direct luminal obstruction [13].

Despite its potential cardiovascular relevance, BAC is inconsistently reported and is generally evaluated using qualitative or manually derived measures [14,15]. The absence of standardized and clinically actionable scoring criteria limits its reproducibility and large-scale integration into cardiovascular risk assessment [14].

Recent advances in artificial intelligence (AI), particularly deep learning, have enabled the automated detection, segmentation, and quantitative assessment of BAC on mammographic images [13,14,15]. Convolutional neural networks and related architectures have demonstrated the feasibility of automated BAC assessment across digital mammography and digital breast tomosynthesis [16,17,18]. Beyond image-level detection, emerging evidence indicates that AI-derived BAC measurements are associated with major adverse cardiovascular events and mortality and may provide prognostic information beyond conventional cardiovascular risk factors or established prediction models [19,20]. Thus, AI may facilitate the transition of BAC from a subjectively reported incidental finding to a standardized and scalable imaging biomarker of cardiovascular risk.

Despite these findings, the evidence remains dominated by retrospective studies with heterogeneous datasets, model architectures, reference standards, and outcome definitions [16,17,18,19]. Prospective clinical validation and standardized thresholds for cardiovascular referral are still lacking, leaving the effects of AI-based BAC assessment on risk reclassification, preventive management, and clinical outcomes uncertain [18,19].

**Figure 1:**
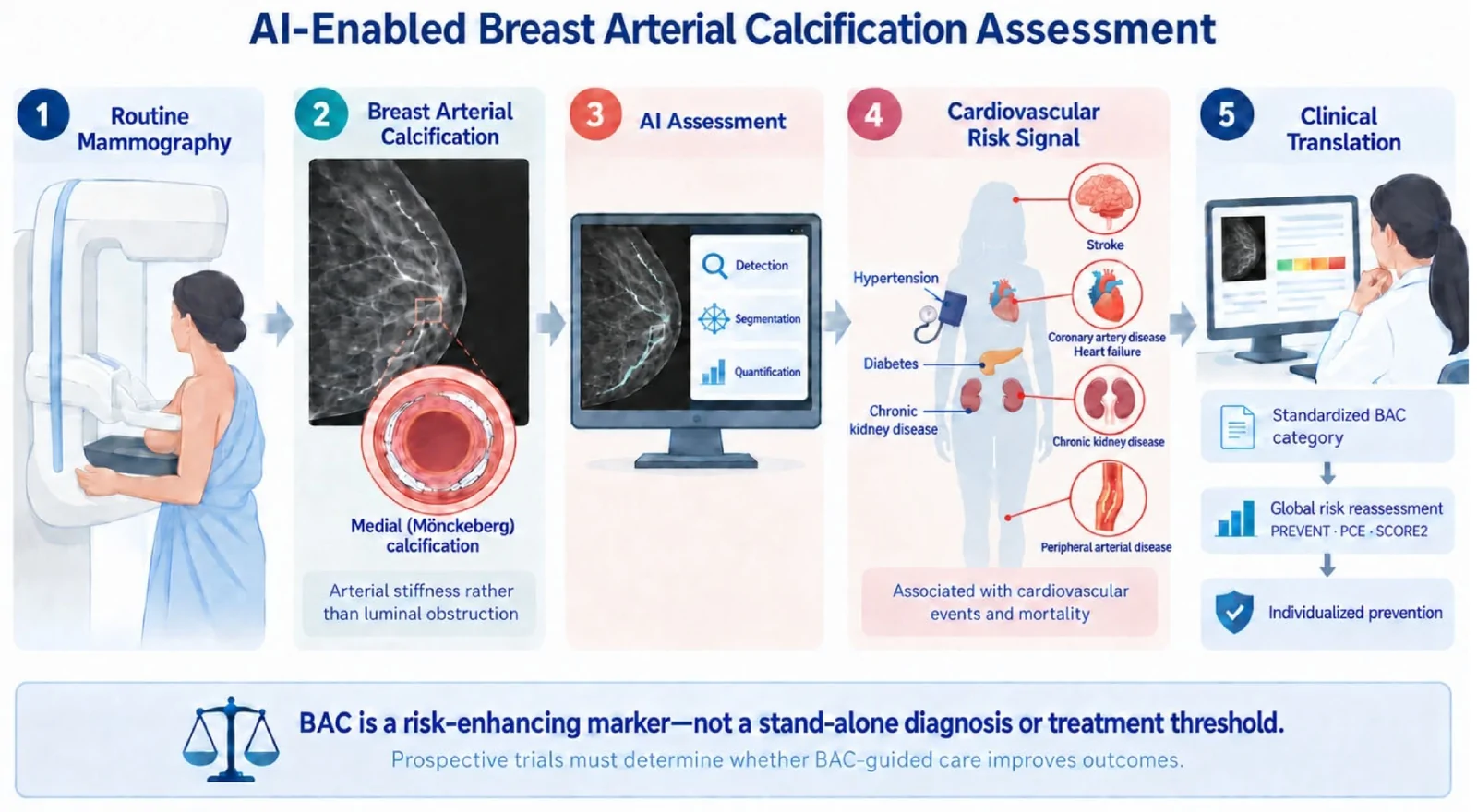
AI-enabled assessment of breast arterial calcification and its potential integration into cardiovascular prevention.

Accordingly, this review critically examines current AI-based methods for detecting, segmenting, and quantifying BAC on mammographic imaging. Particular emphasis is placed on model performance, methodological quality, external validity, prognostic value, integration with established cardiovascular risk prediction tools, and barriers to clinical implementation. We further discuss the steps required to translate automated BAC assessment from retrospective algorithm development into a clinically actionable component of cardiovascular prevention in women. The principal contribution of this narrative review lies in its integration of three levels of evidence that have often been evaluated separately: (1) technical validation of deep-learning methods for BAC detection, segmentation, and quantification; (2) cardiovascular prognostic validation of automatically quantified BAC; and (3) the requirements for clinical translation within mammographic screening and cardiovascular prevention pathways. Figure 1 illustrates this proposed framework, outlining the progression from routine mammography and AI-based BAC assessment to cardiovascular risk evaluation and potential clinical implementation. Current BAC assessment relies largely on visual or semi-quantitative evaluation, which is limited by reader dependence, inconsistent reporting, and the absence of standardized quantitative thresholds. Deep-learning methods offer a potential solution by enabling automated and scalable BAC assessment; however, technical performance alone does not establish cardiovascular prognostic value or clinical utility. This review therefore critically examines the methodological factors that influence the interpretation of reported performance. These include class imbalance, data-partitioning strategies, learning-curve reporting, shortcut learning, external validation, domain shift, and differences between technical and prognostic validation. By integrating these dimensions, this review aims to clarify both the current capabilities of automated BAC assessment and the evidence gaps that must be addressed before it can be incorporated into routine cardiovascular prevention.

## 2. Methodology

This narrative review provides a focused synthesis of the available evidence on artificial intelligence (AI)-based detection, segmentation, and quantification of breast arterial calcification (BAC) on mammographic imaging, along with its potential role in cardiovascular risk assessment. A narrative review design was selected because the available literature encompasses heterogeneous research questions and study types. These include technical algorithm-development studies, segmentation and classification studies, observational cardiovascular outcome studies, systematic reviews, and professional recommendations. These studies differ considerably in populations, imaging modalities, BAC definitions, reference standards, AI tasks, performance metrics, validation strategies, and clinical outcomes. Given this heterogeneity, a single quantitative synthesis was considered inappropriate. The narrative approach therefore enabled the integration of technical, prognostic, and translational evidence while permitting critical appraisal of methodological differences across these domains. A targeted literature search was conducted using PubMed/MEDLINE, Scopus, Web of Science, and Google Scholar. Combinations of the following terms were used: “breast arterial calcification”, “breast artery calcification”, “mammography”, “digital mammography”, “digital breast tomosynthesis”, “artificial intelligence”, “machine learning”, “deep learning”, “convolutional neural network”, “automated detection”, “segmentation”, “quantification”, “cardiovascular risk”, “cardiovascular disease”, “major adverse cardiovascular events”, and “mortality”. Reference lists of relevant articles were also screened to identify additional eligible publications. The search covered English-language publications available from database inception, and the final literature search was performed on 3 September 2026. Both foundational studies on the cardiovascular significance of BAC and contemporary investigations of AI-based image analysis were considered in order to provide an overview of the development and current state of the field. Particular attention was given to studies evaluating algorithmic detection, localization, segmentation, or quantitative measurement of BAC using full-field digital mammography or digital breast tomosynthesis. Investigations reporting diagnostic performance, agreement with radiologist or manually derived assessments, external validation, associations with cardiovascular risk factors, risk reclassification, major adverse cardiovascular events, or mortality were prioritized; relevant systematic reviews, meta-analyses, consensus documents, and professional recommendations were also considered. Studies were considered eligible if they met at least one of the following criteria: (1) evaluation of BAC on mammographic imaging; (2) investigation of AI-based detection, classification, localization, segmentation, or quantitative assessment of BAC; (3) evaluation of associations between BAC and cardiovascular risk factors, cardiovascular disease, major adverse cardiovascular events, or mortality; or (4) provision of clinically relevant evidence regarding cardiovascular risk assessment, reporting, or potential implementation of BAC assessment. Studies were required to provide sufficient methodological information to permit interpretation of the study population, imaging modality, reference standard, AI methodology, or reported clinical outcomes, as applicable. Studies were excluded if they (1) addressed vascular calcification without specific evaluation of BAC, (2) evaluated non-mammographic imaging without direct relevance to BAC assessment, (3) were conference abstracts or other reports without an accessible full-text publication, (4) lacked sufficient methodological or outcome information for meaningful interpretation, or (5) were unrelated to either mammographic BAC assessment or its cardiovascular implications.

Data were synthesized qualitatively because of substantial heterogeneity in study populations, imaging modalities, BAC definitions and reference standards, AI architectures, validation strategies, and reported performance and clinical outcomes. Because this was a narrative rather than a systematic review, no formal risk-of-bias scoring tool was applied. Methodological quality was assessed qualitatively using several study-specific criteria. These included study design and sample size, characterization of the study population and imaging dataset, and clarity and reproducibility of the reference standard or annotation procedure. For AI studies, we additionally considered the separation of training, validation, and test data; the appropriateness of data-partitioning or cross-validation strategies; patient-level separation when multiple images or patches originated from the same individual; and the reporting of training dynamics or learning curves, where available. For studies evaluating cardiovascular prognosis, additional consideration was given to longitudinal follow-up, outcome ascertainment, adjustment for relevant cardiovascular risk factors, and integration with established cardiovascular risk prediction models. The appraisal focused on model development and validation, dataset characteristics, reference annotation methods, task-specific performance, external validity, prognostic value, integration with established cardiovascular risk prediction tools, and feasibility of implementation within breast-screening workflows. The synthesis distinguished between AI detection/classification, segmentation, quantitative BAC measurement, and cardiovascular prognostic assessment, because these represent distinct methodological tasks requiring different validation metrics. When findings were inconsistent, greater weight was given to larger studies, externally validated models, longitudinal outcome data, and evidence supported by systematic reviews or professional guidance. No formal protocol registration or quantitative meta-analysis was undertaken.

## 3. Pathophysiology and Mammographic Characteristics of Breast Arterial Calcification

**Figure 2:**
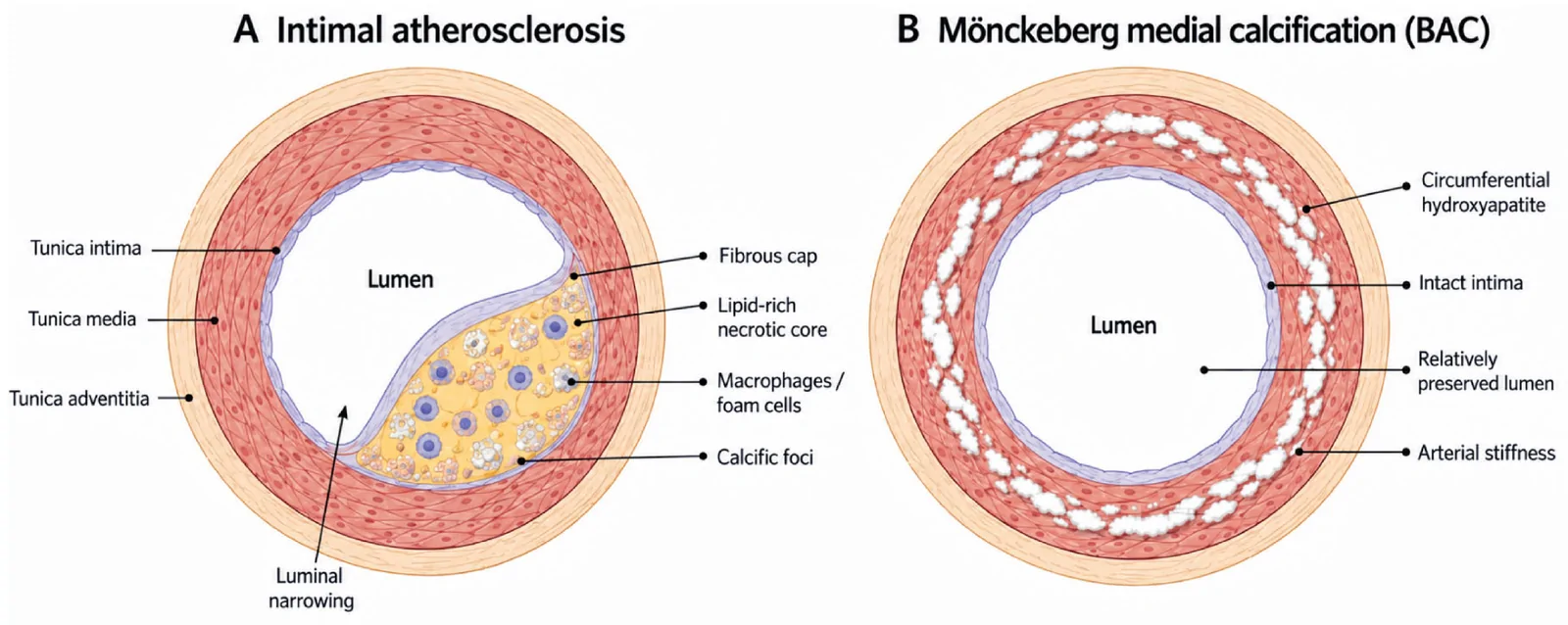
Pathophysiological differences between intimal atherosclerosis and Mönckeberg medial arterial calcification.

Breast arterial calcification predominantly represents a form of Mönckeberg medial calcific sclerosis. This arterial-wall disorder is characterized by calcium deposition within the tunica media—the smooth muscle layer between the vessel’s inner and outer layers. These deposits typically develop circumferentially around the artery, resulting in vascular wall stiffening without atherosclerotic plaque formation, and generally without substantial luminal narrowing [21,22]. Accordingly, Mönckeberg sclerosis is distinct from atherosclerosis: In atherosclerosis, lipids, inflammatory cells, fibrous tissue, and calcium accumulate within the tunica intima, forming plaques that may narrow the arterial lumen or become complicated by rupture and thrombosis. In Mönckeberg sclerosis, the lumen may remain relatively preserved, while the arterial wall loses elasticity and becomes increasingly rigid. This arterial stiffening may increase systolic blood pressure, impose a greater hemodynamic load on the heart, and impair organ perfusion, even in the absence of significant vascular stenosis [23,24]. The principal pathological differences between intimal atherosclerosis and Mönckeberg medial arterial calcification, together with the mechanisms underlying BAC’s development and its characteristic mammographic appearance, are summarized in Figure 2.

On mammography, circumferential calcium deposition along the arterial wall produces two parallel radiopaque lines, commonly described as a “tram-track” or “railroad-track” appearance. When the vessel is visualized in cross-section, the calcification may appear circular or ring-like. In most cases, this characteristic morphology enables the differentiation of benign arterial calcification from microcalcifications associated with malignant breast lesions [21,25].

Occasionally, calcium deposition within the tunica media is incomplete or discontinuous, particularly during the early stages of the process. In such cases, breast arterial calcification may appear as clustered, curvilinear, or branching calcifications that can mimic a pattern suspicious for malignancy [26,27]. Saxena et al. reported such a case, in which histopathologic examination demonstrated that the suspicious mammographic microcalcifications represented discontinuous foci of Mönckeberg medial calcific sclerosis [21]. This presentation is uncommon and should be evaluated according to its mammographic morphology and contemporary diagnostic criteria for breast lesions.

Medial arterial calcification is not a purely passive or degenerative phenomenon but an active, cell-regulated process with features resembling bone formation. Under the influence of aging, inflammation, oxidative stress, and metabolic disturbances, vascular smooth muscle cells may lose their contractile phenotype and acquire an osteogenic profile. These mechanisms provide a biological explanation for the association of breast arterial calcification with aging, diabetes, chronic kidney disease, and disorders of mineral metabolism.

## 4. Mammographic Detection and Conventional Assessment of BAC

In conventional mammographic assessment, BAC is identified visually on standard craniocaudal and mediolateral oblique views [25]. Once its vascular origin has been established based on the characteristic morphology and distribution described above, the finding may be recorded dichotomously as present or absent. Although this binary approach is straightforward and requires little additional interpretation time, it does not capture the extent, density, distribution, or severity of the calcification and may therefore provide limited information for cardiovascular risk stratification [14,28,29]. Several visual and semi-quantitative grading systems have consequently been proposed. These approaches have classified BAC according to the number of affected vessels, calcification density or opacification, total length of the calcified arterial segments, bilaterality, and overall morphological severity [25,30]. One commonly used four-grade system categorizes BAC as absent (Grade 0), few punctate vascular calcifications without coarse or parallel linear calcification (Grade 1), coarse or parallel linear calcification involving fewer than three vessels (Grade 2), or severe calcification involving at least three vessels (Grade 3) [31,32]. Although practical, such ordinal classifications remain dependent on visual interpretation and may not adequately represent the continuous nature of BAC burden. Trimboli et al. proposed the Breast Arterial Calcification Semiquantitative Score (BAC-SS), which combines the number of calcified vessels, the degree of vessel opacification, and the total length of the visible calcified segments on mediolateral oblique mammographic views [28]. The score demonstrated 77% intra-reader and 64% inter-reader reproducibility and required approximately 2 to 3 min per examination. However, identifying individual vascular branches and manually measuring calcified segments remained potential sources of variability, limiting the practicality of this method in high-volume screening programs [28]. Continuous quantitative methods have also been developed to estimate BAC burden more objectively. Densitometric techniques applied to full-field digital mammography can express calcification burden as calcium mass in milligrams, rather than as an ordinal severity category [33].

## 5. Cardiovascular Associations and Prognostic Significance of BAC

### 5.1. Associations with Cardiovascular Risk Factors

Age is the most consistent determinant of BAC, with an almost 3-fold increase in odds for each additional decade of life (pooled OR, 2.98; 95% CI, 2.31–3.85) [11]. Parity, diabetes, hypertension, and chronic kidney disease have also been associated with BAC, consistent with the contribution of reproductive exposure, metabolic dysfunction, and altered mineral metabolism to medial arterial calcification [11,34,35]. A contemporary meta-analysis of 45 studies comprising 68,584 women confirmed associations with diabetes (OR, 1.97; 95% CI, 1.71–2.27), hypertension (OR, 1.82; 95% CI, 1.52–2.18), and, more modestly, hyperlipidemia (OR, 1.24; 95% CI, 1.06–1.45), but it found no significant association with body mass index [10].

These pooled findings are supported by large screening cohorts. Among 17,237 women undergoing screening mammography in a racially and ethnically diverse urban population, Montgomery et al. reported a BAC prevalence of 12.3%, with the likelihood of BAC approximately doubling with each successive decade of life [36]. BAC was less frequent among nulliparous and premenopausal women, as well as among current users of hormone replacement therapy, whereas no association was observed with body mass index [3,36]. In the multiethnic MINERVA cohort of 5059 postmenopausal women, BAC was present in approximately 26% and was more frequent among older women and those with diabetes or prediabetes, hypertension, higher systolic blood pressure, and greater parity [37].

Associations have nevertheless varied across populations. During 23 years of follow-up, Galiano et al. identified hypertension as the principal factor differentiating women with and without BAC, whereas diabetes, dyslipidemia, and obesity did not differ significantly between groups [38]. Smoking history has paradoxically been associated with a lower BAC prevalence in meta-analyses, although the MINERVA cohort found no significant association [8,10,11,37]. This observation should not be interpreted as protective and may reflect residual confounding, selection effects, or survival bias. Overall, BAC appears to reflect a risk profile centered on vascular aging, diabetes, hypertension, reproductive exposure, and disordered mineral metabolism, rather than simply reproducing the conventional risk profile of intimal atherosclerosis.

### 5.2. Association with Coronary Artery Calcification and Coronary Artery Disease

Despite representing distinct vascular processes, BAC and coronary artery calcification (CAC) frequently coexist. Quantitative evidence suggests that BAC is associated not only with the presence of coronary calcium but also with its burden. In 292 women undergoing mammography and chest computed tomography, BAC identified CAC with moderate sensitivity (63%) and specificity (76%), while its discrimination for any CAC was comparable with that of the Framingham Risk Score and Pooled Cohort Equations (area under the curve: 0.73, 0.72, and 0.71, respectively). Moderate-to-severe BAC remained associated with CAC after adjustment for age and hypertension (OR, 3.2), suggesting that BAC conveys vascular information that is not fully captured by conventional risk factors [39].

The association extends beyond coronary calcium to anatomically defined coronary artery disease (CAD), although its magnitude varies according to the population and diagnostic method. Mostafavi et al. reported a moderate correlation between BAC severity and CAD burden on coronary computed tomography angiography (CCTA) (r = 0.48; *p* < 0.001), with moderate-to-severe CAD present in 10 of 12 women with moderate or severe BAC [40]. Similarly, in the SCOT-HEART sub-study, BAC was associated with CAC and obstructive CAD among women evaluated for suspected stable angina [30]. These findings support BAC as a marker of an adverse coronary phenotype but do not establish that medial calcification of the breast arteries directly reflects the extent of intimal coronary atherosclerosis.

Meta-analytic evidence reinforces this distinction. Across 31 studies including 35,583 women, BAC was associated with approximately 2.6-fold higher odds of CAD, with the strongest association observed when CAD was assessed using computed tomography (OR, 3.90; 95% CI, 2.53–6.03) [41]. Greater BAC severity appeared to carry more information than its presence alone: moderate-to-severe BAC was associated with CAD, whereas estimates for mild BAC were not statistically significant. An earlier analysis restricted to angiographically defined disease also demonstrated an association (OR, 3.86; 95% CI, 3.25–4.59) [42]. However, these estimates should be interpreted cautiously, because most contributing studies were cross-sectional, included symptomatic or clinically selected patients, used heterogeneous definitions of BAC and CAD, and demonstrated substantial between-study heterogeneity. Evidence of publication bias further limits the certainty of the pooled effect [41].

BAC also has limited value as a rule-out marker. In a contemporary CCTA cohort, correlations of BAC severity with CAC and CAD-RADS categories were weak, and 18.2% of women without BAC nevertheless had obstructive CAD. The absence of BAC was associated with a low prevalence of severe CAC only among women younger than 55 years and did not exclude obstructive disease or high-risk coronary plaque [43]. Consequently, BAC should not be considered a surrogate for CAC scoring, CCTA, or clinically indicated ischemia testing.

Taken together, the current evidence supports BAC as a risk-enhancing imaging marker rather than a diagnostic test for coronary atherosclerosis: its presence, particularly when extensive, identifies a population with a higher probability of concomitant coronary disease and may justify reassessment of global cardiovascular risk. However, incomplete concordance with CAC, coronary stenosis, and high-risk plaque indicates that BAC provides complementary rather than interchangeable information. Whether this information meaningfully improves risk classification and clinical decision-making beyond established prediction models remains to be determined prospectively.

### 5.3. Incident Cardiovascular Events and Mortality

Overall, BAC has been associated with an increased risk of incident cardiovascular events and mortality, supporting its potential role as a marker of systemic vascular and cardiometabolic disease [8,10,44,45]. Within the cardiovascular–kidney–metabolic framework, BAC may reflect cumulative metabolic and renal vascular injury rather than the presence of individual cardiovascular risk factors alone [46,47]. Among women with diabetes, BAC prevalence approached 40% and was associated with longer diabetes duration and a greater burden of microvascular complications, suggesting a relationship with cumulative glycemic exposure [48]. A longitudinal study further demonstrated accelerated BAC progression with worsening kidney dysfunction, reaching 8.1 mm per breast per year among patients with an estimated glomerular filtration rate below 40 mL/min/1.73 m^2^ and approximately 20 mm per breast per year in end-stage kidney disease [49]. After kidney transplantation, progression decreased to approximately the rate observed in control participants, supporting an association between BAC progression and the metabolic and mineral disturbances accompanying advanced kidney disease [49]. Although these findings position BAC as a potential integrative marker of cardiovascular–kidney–metabolic (CKM) burden, current observational evidence does not establish its role in CKM staging or treatment selection.

BAC has also been associated with peripheral artery disease (PAD), although the available evidence is derived from heterogeneous populations. Among postmenopausal women, BAC was independently associated with an ankle–brachial index below 0.90 (OR, 1.37; *p* = 0.04) [37]. A stronger association was reported among women with end-stage kidney disease (OR, 4.56; *p* < 0.001), but the highly selected population limits generalizability to women undergoing routine mammographic screening [50]. In the prospective EPIC-NL cohort, severe BAC was associated with incident PAD after multivariable adjustment (HR, 3.24; 95% CI, 1.16–9.00), although the wide confidence interval indicates limited precision [51]. Collectively, these findings support BAC as a marker of systemic arterial injury, but they do not establish its diagnostic accuracy as a screening test for PAD.

BAC has also been associated with cerebrovascular events. A 2026 meta-analysis of 10 cohort studies comprising 52 413 women found that BAC was associated with an approximately 2-fold higher risk of stroke (RR, 2.09; 95% CI, 1.58–2.75), although between-study heterogeneity was moderate (I^2^ = 64.4%) [52]. This finding is consistent with an earlier cohort of 12 761 women, in which BAC was independently associated with ischemic stroke over a median follow-up of 24.8 years (HR, 1.41; 95% CI, 1.11–1.78) [53]. However, inconsistent BAC definitions and the inability of several studies to distinguish ischemic from hemorrhagic stroke limit clinical interpretation; BAC should therefore be considered a marker of increased cerebrovascular risk rather than a stroke-specific screening test. Evidence across other cardiovascular outcomes is less uniform: In a meta-analysis of nine cohorts comprising 46 958 women, BAC was associated with heart failure (RR, 2.14; 95% CI, 1.38–3.32), composite atherosclerotic cardiovascular disease (ASCVD) (RR, 1.58; 95% CI, 1.23–2.04), cardiovascular mortality (RR, 2.94; 95% CI, 1.32–6.54), and all-cause mortality (RR, 2.04; 95% CI, 1.08–3.84) [10]. Conversely, the pooled association with myocardial infarction was not statistically significant (RR, 1.54; 95% CI, 0.89–2.66) and was sensitive to the exclusion of a single study [10]. Thus, BAC appears to identify global vascular vulnerability more consistently than myocardial infarction risk specifically [45].

## 6. Artificial Intelligence-Based Assessment of Breast Arterial Calcification

Manual BAC assessment is time-consuming, reader-dependent, and insufficiently standardized for population-level use. AI enables automated detection, segmentation, and quantification, although accurate BAC classification does not necessarily ensure reliable measurement of calcification burden [54,55,56].

### 6.1. Automated Detection, Segmentation, and Quantification

Artificial intelligence-based BAC assessment includes three distinct tasks: detection of BAC, segmentation of calcified arterial segments, and quantification of total calcification burden. Early classification models established that BAC could be identified directly on mammograms, whereas subsequent U-Net-derived architectures enabled pixel-level delineation of its thin, branching morphology [57,58]. For example, SCU-Net achieved a Dice score of 0.729 and a Jaccard index of 0.581, with automated measurements closely correlating with manual annotations [16]. A recurrent attention U-Net subsequently achieved a sensitivity of 69.6%, an F1 score of 0.666, and a Jaccard index of 0.595 [59]. Although its reported pixel-level accuracy was 99.9%, this metric should be interpreted cautiously because BAC occupies only a small proportion of mammographic pixels, and correct classification of background tissue may inflate overall accuracy [59]. Transparent reporting of training and validation dynamics is also informative in this setting. Severe foreground–background imbalance may produce apparently favorable optimization while providing limited information about minority-class segmentation. Learning curves can help identify convergence and train–validation divergence, but they should be interpreted together with BAC-sensitive overlap metrics and performance on held-out data. Stable learning curves alone cannot establish that a model has learned BAC-specific features rather than dataset-specific shortcuts [60,61]. BAC segmentation presents additional technical challenges because calcified breast arteries are typically thin, elongated, and branching structures that occupy only a small fraction of the mammographic image. This morphology creates marked foreground–background class imbalance and increases the risk of missing small or discontinuous calcified segments. Class imbalance is a recognized challenge in medical image segmentation, particularly when the target structure occupies only a small proportion of the image, and the choice of loss function can substantially influence model optimization and segmentation performance [62]. Loss functions based on cross-entropy, Dice overlap, focal weighting, or combinations of these approaches may therefore be considered depending on the degree and characteristics of the imbalance. Image resolution and pre-processing are similarly important, because downsampling may result in the loss of fine spatial information that cannot be fully recovered during decoding—a recognized limitation of U-Net-based medical image segmentation [62,63]. Annotation quality also influences apparent model performance, as manual delineation of thin or discontinuous calcifications may introduce inter-observer variability into the reference standard. Post-processing can reduce isolated false-positive detections or improve structural continuity but may also remove small true-positive regions. Finally, differences in detector characteristics, acquisition protocols, image-processing pipelines, vendors, and patient populations may introduce domain shift and reduce performance when a model is applied outside its development environment. These generalization challenges are well recognized across U-Net-based medical image segmentation and reinforce the importance of external, multivendor validation [62,63]. Overlap-based metrics, sensitivity, false-positive burden, and performance stratified by BAC severity are therefore more informative for evaluating segmentation models.

Case-level classification and pixel-level segmentation represent distinct tasks and should be evaluated using task-specific performance metrics. In 1493 women, a VGG16-based model achieved an area under the receiver operating characteristic curve (AUC) of 0.95 and localized BAC in close agreement with manually measured calcification length (Spearman ρ = 0.88) [17]. However, related pretrained networks achieved AUCs of only 0.77 to 0.79 in an independent test dataset, indicating that performance is sensitive to dataset composition and training strategy [18]. Multitask and semi-supervised methods may reduce annotation requirements by incorporating anatomical information and unlabeled examinations, but their evaluation has remained predominantly retrospective and based on limited validation samples [64,65,66]. Furthermore, radiologist-generated annotations reproduce expert interpretation rather than providing an independent biological reference standard. In an external cohort, an automated BAC score correlated with manual grading but demonstrated limited discrimination for marked coronary artery calcification (AUC, 0.64), emphasizing that accurate BAC measurement and identification of coronary atherosclerosis represent distinct validation targets [66].

Although most automated BAC models have been evaluated using digital mammography or synthesized two-dimensional images, applicability to digital breast tomosynthesis is necessary for integration into contemporary screening programs. Watanabe et al. evaluated an AI system on 314 digital mammography examinations and 277 digital breast tomosynthesis examinations, reporting similar discrimination for BAC detection with digital mammography (AUC, 0.96; sensitivity, 87%; specificity, 92%) and digital breast tomosynthesis (AUC, 0.95; sensitivity, 88%; specificity, 90%) [56]. These findings support the feasibility of cross-modality detection but should not be interpreted as evidence that quantitative BAC measurements are interchangeable across imaging platforms. The study was retrospective, assessed binary detection rather than calcification burden, and used radiologist consensus as the reference standard. Further multivendor validation is therefore required before automated BAC scores can be compared longitudinally or applied uniformly across screening programs.

Collectively, the available studies demonstrate the technical feasibility of automated BAC assessment but provide insufficient evidence for routine clinical implementation. Before automated reporting can inform cardiovascular prevention, algorithms require multicenter and multivendor validation, assessment across demographic and clinical subgroups, standardized quantitative thresholds, and prospective evaluation of risk reclassification. Interpretation of reported AI performance should also account for the unit of analysis, mammographic views and acquisition systems, annotation or reference standard, data-partitioning strategy, and the presence or absence of independent external validation, as these factors directly influence the risk of overfitting and the transportability of model performance across institutions. The appropriateness of the validation strategy should also be considered in relation to dataset size and structure. In larger datasets, patient-level separation into training, validation, and held-out test sets can provide a direct estimate of performance on unseen observations, whereas appropriately designed cross-validation may provide more stable performance estimates when development datasets are small. Regardless of the strategy used, partitioning should occur at the patient level whenever multiple images, views, or patches originate from the same individual to minimize information leakage. Performance estimates derived from very small validation or test samples should also be interpreted cautiously because of their greater statistical uncertainty. Most importantly, clinical utility should be defined by whether AI-derived BAC assessment changes preventive management and improves cardiovascular outcomes—not solely by its agreement with manual image annotation. Reporting of training dynamics was inconsistent across studies: explicit epoch-wise learning curves were infrequently presented, although several studies described related procedures such as validation-based checkpointing, early stopping, cross-validation, or staged training. Where learning curves were available, they provided additional information regarding model optimization and potential overfitting; however, their absence in most published reports limits retrospective assessment of training convergence and train–validation divergence. More transparent reporting of epoch-wise training and validation performance should therefore be encouraged in future BAC studies. Principal studies evaluating the technical performance of AI-based BAC detection, segmentation, and quantification are summarized in Table 1, whereas studies evaluating the cardiovascular prognostic value of AI-quantified BAC are summarized separately in Table 2.

### 6.2. Prognostic and Clinical Value of AI-Quantified BAC

The clinical relevance of automated BAC assessment depends on whether quantitative burden predicts cardiovascular outcomes beyond conventional risk estimation. In an early retrospective study of 99 women, Rose et al. found that participants who experienced major adverse cardiovascular events (MACEs) had higher mean AI-derived BAC scores than those without such events (35.3 vs. 15.1; *p* = 0.01) [55]. BAC remained associated with MACEs after adjustment for age and ASCVD risk (OR per score unit, 1.02; 95% CI, 1.00–1.04). However, the small sample (99 women, including 26 with MACEs) and the absence of time-to-event analysis limit the precision and generalizability of this finding [55].

Subsequent studies in larger populations demonstrated a consistent dose–response association between automated BAC burden and adverse outcomes. In 18,092 women followed for a median of 6 years, Allen et al. reported higher mortality among women with BAC than among those without BAC (7.8% vs. 2.3%), as well as a higher incidence of a composite of myocardial infarction, heart failure, stroke, or death (12.4% vs. 4.3%) [19]. Adjusted risks increased progressively across BAC quartiles, suggesting that quantitative burden provides more prognostic information than binary detection alone. Similarly, Ibrahim et al. found progressively higher adjusted MACE risk across mild, moderate, and severe area-based BAC categories (HRs: 1.13, 1.30, and 1.58, respectively) [65]. Nevertheless, adding BAC grading to conventional risk factors produced minimal improvement in 5-year discrimination (AUC: 0.645 to 0.646), illustrating that an independent association with outcome does not necessarily translate into meaningful incremental prediction.

More recent multicenter analyses have examined whether automated BAC can identify residual risk not captured by established prediction models. Among 21 514 women without known cardiovascular disease, Nerlekar et al. found that age-adjusted BAC burden remained associated with MACEs across ASCVD risk categories [67]. Among women classified as low risk, BAC below and above the age-specific median was associated with adjusted HRs of 1.66 and 2.31, respectively. These findings suggest that interpreting BAC relative to age may identify women whose risk is underestimated by conventional short-term assessment. In a multicenter analysis of 123,762 women from two healthcare systems, Dapamede et al. demonstrated a graded association between automated BAC area and MACEs after adjustment for PREVENT scores [20]. Compared with the absence of BAC, risk was approximately 30% higher with mild BAC, 75% higher with moderate BAC, and 2.8- to 3.3-fold higher with severe BAC across the two cohorts. Associations among women younger than 50 years further suggest that BAC may reveal vascular risk before conventional estimates become elevated [20]. Collectively, AI-quantified BAC demonstrates consistent prognostic associations but remains insufficiently validated to guide clinical decisions. Prospective studies must determine whether BAC-guided risk assessment improves preventive management and cardiovascular outcomes without increasing unnecessary testing or treatment. As summarized in Table 2, evidence for a dose–response association between AI-quantified BAC and cardiovascular events is increasingly consistent, whereas evidence of incremental clinical utility remains limited.

### 6.3. Reporting, Clinical Integration, and Management Pathways

Clinical integration of AI-derived BAC requires more than automated measurement—it requires standardized reporting, clear responsibility for follow-up, and evidence-based management pathways. Current practice remains inconsistent, and surveys of radiologists have identified substantial variation in whether BAC is reported, how severity is described, and whether cardiovascular assessment is recommended [14,15]. Similar uncertainty exists among referring clinicians: in a survey of 186 mammography-ordering physicians, 79.6% indicated that reported BAC would prompt further evaluation of coronary disease or cardiovascular risk factors; however, the low response rate and reliance on hypothetical decisions limit conclusions regarding actual changes in care [32].

Patient notification may facilitate communication across specialties. In a prospective study of 494 women undergoing mammography, Vincoff et al. identified BAC in 68 participants; among the 42 who completed follow-up, 57.1% reported discussing the finding with a primary care clinician or cardiologist [68]. Although most respondents considered the information helpful, the study did not demonstrate changes in risk-factor control, preventive treatment, or cardiovascular outcomes. Similarly, a primary care study found that BAC identified women with higher estimated cardiovascular risk and suggested that approximately one-third of BAC-positive participants might warrant additional evaluation, but it assessed potential rather than observed changes in management [69].

The BEACON study further evaluated the feasibility of combining breast cancer screening with opportunistic cardiovascular assessment, illustrating a possible pathway linking mammography findings to cardiovascular risk evaluation [31]. However, feasibility and clinician acceptance should not be considered evidence of clinical effectiveness. Before routine implementation, standardized BAC categories must be linked to prespecified actions and tested prospectively against usual care.

A pragmatic pathway would incorporate the AI-derived BAC category into the mammography report and electronic health record, with follow-up assigned to the clinician responsible for cardiovascular prevention. In asymptomatic women, moderate or severe BAC should prompt review of blood pressure, lipid and glycemic status, smoking, kidney function, and other relevant risk modifiers, followed by estimation of global cardiovascular risk using PREVENT, the Pooled Cohort Equations, or SCORE2/SCORE2-OP, according to the population and clinical setting [70,71,72,73,74]. BAC may be most informative among women classified as having low or intermediate risk, in whom it could identify untreated risk factors or support shared preventive decision-making [8,20,67].

**Figure 3:**
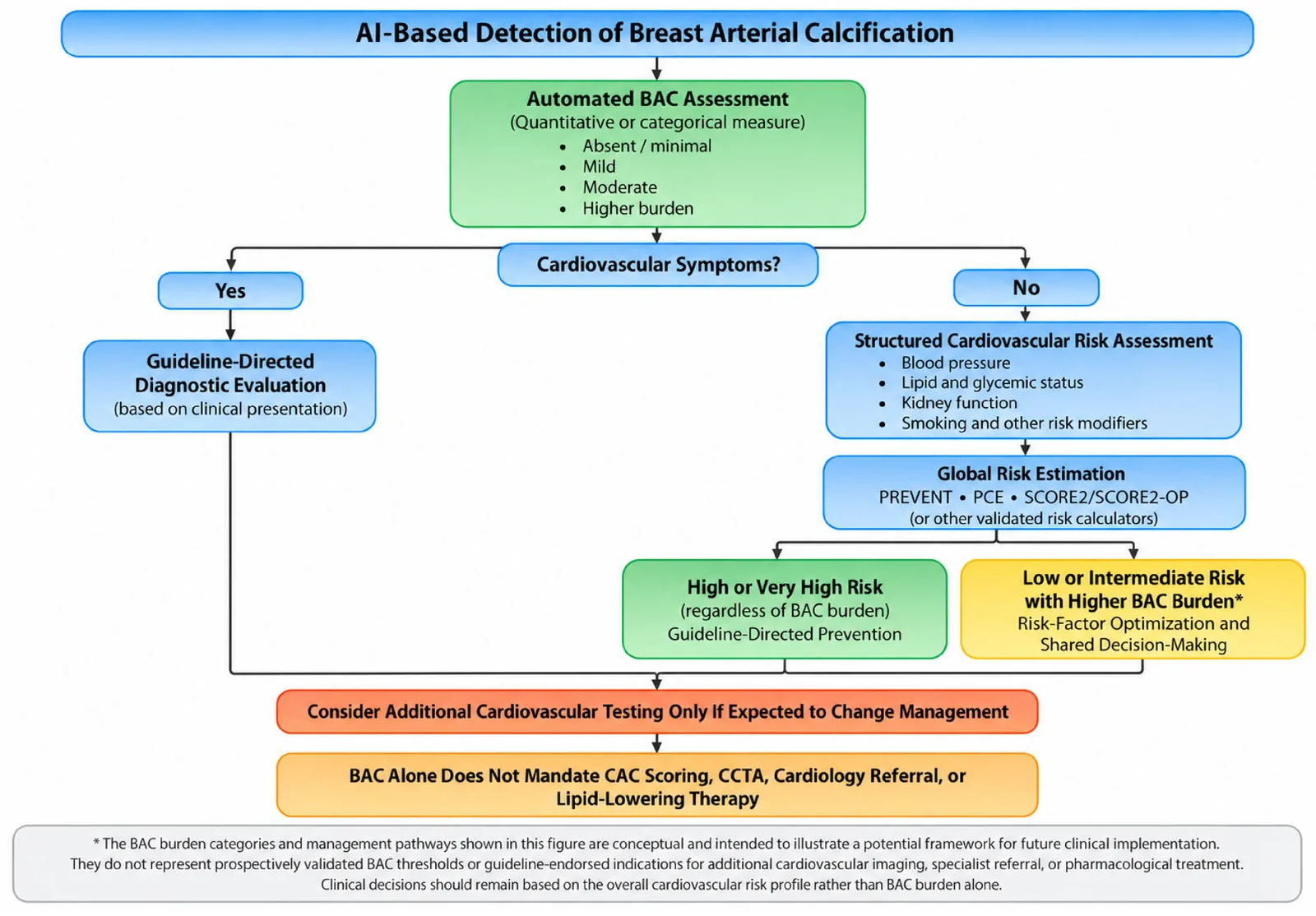
Proposed clinical pathway following artificial intelligence-based detection of breast arterial calcification on screening mammography. **Abbreviations:** AI, artificial intelligence; BAC, breast arterial calcification; CAC, coronary artery calcium; CCTA, coronary computed tomography angiography; PCE, Pooled Cohort Equations; PREVENT, Predicting Risk of Cardiovascular Disease EVENTs; SCORE2, Systematic Coronary Risk Evaluation 2; SCORE2-OP, Systematic Coronary Risk Evaluation 2–Older Persons.

BAC alone should not mandate coronary artery calcium scoring, coronary computed tomography angiography, cardiology referral, or lipid-lowering therapy. These decisions should remain guided by symptoms, estimated global risk, guideline-defined treatment eligibility, and whether further testing is expected to change management. Thus, AI-derived BAC should currently be regarded as a risk-enhancing signal that prompts structured cardiovascular reassessment rather than as an independently actionable treatment threshold. Prospective implementation trials are required to determine whether BAC-guided care improves preventive management and cardiovascular outcomes without increasing unnecessary testing, cost, overtreatment, or patient anxiety. Importantly, the proposed BAC categories and downstream management pathways are conceptual rather than validated clinical thresholds and should not be interpreted as guideline-based indications for additional testing, referral, or treatment. A proposed framework for cardiovascular risk assessment and subsequent clinical management following AI-based detection of BAC on screening mammography is presented in Figure 3.

## 7. Current Evidence Gaps and Future Research Priorities

### 7.1. Standardization and External Validation

The absence of a standardized BAC definition remains a major barrier to clinical translation. Published studies have used binary detection, ordinal visual grades, calcified-vessel counts, segment length, calcium mass, area-based measurements, and proprietary continuous scores, with inconsistent thresholds for mild, moderate, and severe disease [13,14,25,28,33]. Consequently, apparently similar BAC categories may represent different calcification burdens across studies and algorithms. Consensus is needed regarding the mammographic views to be analyzed, aggregation of bilateral findings, minimum detectable lesion, quantitative unit, and clinically interpretable severity categories. Standardization must extend beyond measurement to algorithm validation. Most automated models were developed retrospectively using selected datasets and radiologist-generated annotations, with limited independent, multicenter, and multivendor evaluation [16,17,18,56,57,58,59,64]. Performance should be assessed separately across full-field digital mammography, synthesized two-dimensional mammography, and digital breast tomosynthesis, because agreement within one imaging modality does not establish interchangeability across platforms. Future studies should report patient-level data separation, calibration, false-positive burden, performance according to BAC severity, and prespecified failure analyses, rather than relying predominantly on overall accuracy or internal cross-validation. Future studies should report patient-level data separation, calibration, false-positive burden, performance according to BAC severity, and prespecified failure analyses, rather than relying predominantly on overall accuracy or internal cross-validation [75].

### 7.2. Generalizability, Bias, and Algorithmic Transparency

The generalizability of existing BAC algorithms is uncertain because many development cohorts were drawn from single institutions or clinically selected populations and incompletely characterized with respect to race and ethnicity, socioeconomic status, breast density, reproductive history, and cardiometabolic disease. Because BAC prevalence increases markedly with age, algorithms trained using enriched BAC-positive samples may preferentially recognize extensive calcification and perform less reliably in younger women or those with subtle disease. This spectrum effect is clinically important because women with initially low or intermediate estimated risk may represent the population in whom BAC provides the greatest incremental information [8,20,67]. Validation should therefore include subgroup-specific discrimination, calibration, and error analyses across age groups, racial and ethnic populations, mammographic density categories, imaging vendors, and relevant comorbidities [76]. Explainability methods may help identify whether algorithms appropriately localize breast arteries rather than nonvascular calcifications, but visual heatmaps alone do not establish biological validity or eliminate bias. Post-deployment surveillance will also be required to detect performance drift following changes in imaging equipment, acquisition protocols, or screening populations. Reporting of training dynamics represents an additional consideration when evaluating deep-learning models for BAC assessment. Training and validation learning curves can provide useful evidence regarding model convergence and may reveal patterns suggestive of overfitting, such as continued improvement in training performance accompanied by stagnation or deterioration in validation performance. Conversely, parallel stabilization of training and validation loss or performance may support appropriate model optimization. However, learning curves should be interpreted as one component of model appraisal rather than as definitive evidence of biologically meaningful learning, because apparently satisfactory convergence does not exclude reliance on spurious or dataset-specific features [60,61]. Transparent reporting of training and validation dynamics, together with appropriately separated held-out testing and independent external validation, therefore provides a more comprehensive assessment of model robustness and generalizability. A distinct but related concern is shortcut learning, whereby a model achieves apparently high performance by exploiting features that correlate with the target label within the development dataset but are not biologically related to the target itself. In medical imaging, such shortcuts may arise from institution- or scanner-specific characteristics, acquisition and pre-processing artifacts, patient-positioning patterns, embedded image markers, or demographic and other dataset-specific proxies [61,77,78]. These dependencies may remain undetected during conventional internal validation when training and validation datasets share the same acquisition environment. Consequently, apparently strong internal performance may deteriorate when the model is applied to a different clinical population or imaging system. Importantly, satisfactory training and validation learning curves do not exclude shortcut learning when the same spurious association is present in both datasets. Robustness and sensitivity analyses, ablation experiments, interpretable localization methods, subgroup analyses, and evaluation across genuinely independent institutions, populations, and acquisition systems therefore provide complementary evidence for assessing whether model performance depends on dataset-specific features [60,61,78]. External validation itself should also be interpreted cautiously, because datasets labeled as “external” may remain closely related to the development distribution. External cohorts may still share important characteristics with the training population, including scanner manufacturers, acquisition and pre-processing protocols, geographic or referral characteristics, temporal periods, and demographic profiles. Such similarities may limit the degree of true distributional independence. Evidence from radiologic deep-learning studies indicates that performance frequently decreases when models are evaluated outside their development environment: a systematic review of externally validated radiologic deep-learning algorithms found that 81% showed some deterioration in external performance, with 24% demonstrating a substantial decrease [79]. Cross-institutional studies have similarly demonstrated that models may exploit institution-specific characteristics and perform differently when transferred between health systems [77,79]. More broadly, systematic evidence on medical-image classification has identified acquisition-related covariate shift and population differences as important barriers to model generalization [80]. Therefore, external validation should be characterized not only by whether the test dataset originates from a nominally separate cohort but also by its geographic, temporal, demographic, institutional, and technical independence from the development data. For BAC algorithms, stronger evidence of transportability would require evaluation in genuinely independent populations from different institutions and acquisition environments, ideally accompanied by subgroup-specific performance and calibration analyses. A complementary cross-domain methodological perspective is provided by quantitative imaging phenotyping in computational histopathology. Histomics research has highlighted that quantitative imaging phenotypes may depend on segmentation quality, feature stability, acquisition and pre-processing variability, and the interpretability of derived representations, while domain shift across datasets and institutions may further compromise reproducibility [81]. Although these observations arise from histopathology rather than mammography and should not be interpreted as direct evidence regarding BAC, they illustrate broader methodological principles relevant to quantitative imaging biomarkers. Applied to AI-derived BAC phenotypes, these considerations reinforce the need to evaluate the stability of quantitative measurements across segmentation methods, acquisition systems, vendors, and populations, as well as to establish that derived features remain interpretable and reproducible before they are considered clinically transportable.

### 7.3. Prospective Clinical Utility and Outcome Evaluation

The principal unresolved question is whether automated BAC reporting improves patient care. Existing studies demonstrate associations with cardiovascular outcomes, but most are retrospective and do not establish that BAC-guided management improves risk-factor control, treatment selection, or cardiovascular prognosis [19,20,65]. Similarly, surveys and notification studies indicate that radiologists, referring clinicians, and patients are generally receptive to BAC reporting, but acceptance and feasibility do not constitute evidence of clinical benefit [15,28,31,32,39].

Prospective implementation studies should compare standardized BAC reporting with usual care and evaluate prespecified outcomes, including completion of cardiovascular risk assessment, identification of previously untreated hypertension, diabetes or dyslipidemia, initiation or intensification of guideline-directed prevention, use of downstream imaging, patient anxiety, healthcare costs, and cardiovascular events. Such studies should also determine whether BAC meaningfully improves discrimination, calibration, and treatment-relevant risk reclassification when added to PREVENT, PCE, SCORE2, or SCORE2-OP [71,73,74]. Randomized or pragmatic cluster trials may ultimately be required to establish whether BAC-guided prevention improves outcomes without causing disproportionate testing or overtreatment. Clinical implementation requires interoperable reporting, clearly assigned follow-up responsibility, and validated action thresholds, with success determined by whether acting on AI-derived BAC findings improves patient outcomes rather than by detection performance alone.

## 8. Limitations

This review has several limitations. First, it was designed as a narrative synthesis and did not use a prospectively registered protocol, formal risk-of-bias assessment, or quantitative meta-analysis. Although multiple databases and reference lists were searched, the targeted search strategy and restriction to English-language publications may have resulted in the omission of relevant studies. Comparisons across studies were further limited by substantial heterogeneity in study populations, mammographic modalities, BAC definitions, reference standards, AI architectures, validation strategies, and cardiovascular outcomes. In addition, this is a rapidly evolving field, and several recent algorithms and outcome studies have undergone limited independent replication. Accordingly, conclusions regarding comparative model performance, clinically actionable BAC thresholds, and the effects of BAC-guided management should be interpreted cautiously.

## 9. Conclusions

Breast arterial calcification is a distinct manifestation of medial arterial calcification that can be identified opportunistically during routine mammographic screening. Available evidence consistently associates BAC with adverse cardiovascular risk profiles and outcomes in women. Deep-learning methods have demonstrated the technical feasibility of automated BAC detection, segmentation, and quantification, potentially overcoming important limitations of visual and manually derived assessment. However, reported performance varies according to the computational task, dataset composition, reference standard, validation strategy, and acquisition environment, and the available evidence remains limited by inconsistent external validation, heterogeneous quantitative definitions, and incomplete assessment of model robustness and generalizability. Automated BAC burden has also demonstrated graded associations with major adverse cardiovascular events and mortality, including among women classified as having low cardiovascular risk by conventional prediction models. Nevertheless, prognostic association should be distinguished from incremental predictive and clinical utility; available studies have not established that incorporating BAC into cardiovascular risk assessment improves treatment decisions or patient outcomes.

Accordingly, AI-derived BAC should currently be regarded as a risk-enhancing imaging signal that may support structured cardiovascular risk reassessment, rather than as an independently actionable indication for additional imaging, specialist referral, or pharmacological treatment. Clinical translation will require standardized BAC definitions and quantitative thresholds, genuinely independent multicenter and multivendor validation, transparent reporting of model development and failure modes, and prospective implementation studies evaluating risk reclassification, downstream testing, preventive management, cost, and cardiovascular outcomes. The ultimate value of automated BAC assessment will therefore depend not only on accurate image analysis but also on whether its integration into mammographic screening leads to measurable improvements in cardiovascular prevention without unnecessary testing or overtreatment.

## Figures and Tables

**Table 1:** Technical validation of artificial intelligence-based detection, segmentation, and quantification of breast arterial calcification.

Study	AI Task and Model	Dataset, Unit of Analysis, and Imaging	Reference Standard	Training/Validation/Test Strategy	External Validation	Primary Task-Specific Metric(s)	Critical Interpretation	Learning-Curve Reporting
Ibrahim et al. 2026 [65]	Segmentation and severity grading; semi-supervised U-Net with progressive pseudo-labeling	2560 annotated single-view mammograms from 1280 screening examinations; 1318 CC and 1242 MLO views; 11 scanner types from 7 vendors; additional 6000 unlabeled images	Manual segmentation reviewed and confirmed by 2 senior radiologists; severity grading additionally compared with 3-radiologist CSBI consensus	2278 annotated images used for training/validation; 282 images (141 bilateral examinations) held out for final testing; patient-level separation; pseudo-labeling restricted to training data	Multivendor, but no independent external cohort	Segmentation: JSC 0.614; F1 0.756; recall 0.764. Severity grading: weighted κ vs. consensus 0.90; Grade 3 AUC 0.93, sensitivity 0.80, specificity 0.93	Broad multivendor development improves assessment of cross-system robustness; however, the expert grading comparison involved only 20 examinations, and independent external validation remains required	Not reported; validation-loss early stopping used
Watanabe et al. 2025 [56]	Binary BAC detection; commercial deep convolutional neural network (CNN) (cmAngio)	Test sets: 253 women/314 DM examinations/1262 images and 143 women/277 DBT examinations/1229 projections; Hologic systems	Majority consensus of 3 experienced breast radiologists with manual BAC markings	Algorithm trained separately on large multisite DM and DBT datasets; retrospective test sets from 6 US institutions with patient-level separation from training data	Independent multisite test cohort; test images single-vendor (Hologic)	Case-level detection: DM AUC 0.96, sensitivity 87%, specificity 92%; DBT AUC 0.95, sensitivity 88%, specificity 90%	Demonstrates strong case-level binary detection across DM and DBT; does not validate quantitative BAC burden or cardiovascular prognostic performance	Not reported; commercial pretrained model
Saccenti et al. 2025 [66]	BAC detection and quantitative scoring; commercial deep-learning software (iCAD)	502 women with digital mammography and thoracic CT; bilateral CC and MLO views; Siemens Inspiration mammography	12-point manual BAC score by radiologist; CAC independently graded on CT using a 12-point scale	AI previously trained on mammograms from 2800 patients across 8 US centers; no overlap with study cohort	Yes—independent external retrospective cohort	BAC quantification: correlation with manual BAC score r = 0.83; BAC detection AUC 0.87; marked-BAC AUC 0.89. Marked CAC discrimination: AUC 0.64, sensitivity 32.7%, specificity 96.1%	Strong external agreement with manual BAC scoring, but BAC quantification and discrimination of marked coronary calcium represent distinct validation targets; marked-CAC sensitivity was low	Not reported; commercial pretrained model
AlJabri et al. 2024 [59]	Pixel-level segmentation and BAC quantification; recurrent attention U-Net	1436 synthesized 2D CC/MLO images retained from an initial 2000-image dataset; DBT acquired on 6 Hologic Dimensions units at 3 centers	BAC boundaries manually delineated by trained annotators and reviewed/modified by an expert breast radiologist to establish gold-standard masks	1149 images training (80%), 143 validation (10%), 144 testing (10%); non-overlapping splits	No independent external validation	Segmentation: sensitivity 69.6%; F1 0.666; Jaccard 0.595; pixel accuracy 99.9%. Quantification: maximum R^2^ 0.83 vs. ground-truth masks	Pixel accuracy is inflated by extreme class imbalance (BAC ≈ 0.2% of pixels); overlap metrics are more informative. Evaluation remained restricted to synthesized 2D images from Hologic systems	Partial; validation-based model selection, no paired curves
Mobini et al. 2024 [18]	Binary BAC classification/localization; comparison of 11 pretrained CNNs	1493 women; 5972 four-view FFDM images; Giotto IMAGE 3D/TOMO systems (IMS)	Expert image-level BAC/non-BAC labels; disagreements resolved by consensus; manual BAC-length measurements used for localization comparison	Patient-level 70%/15%/15% train/validation/test split; final sets after training undersampling: 1640/888/916 images	No—single-center, single-manufacturer dataset	Classification: test AUC VGG16 0.79, MobileNet 0.78, DenseNet201 0.77; highest test F1 0.53 (VGG16). Localization: VGG16 Spearman ρ = 0.68 with manual BAC length	Independent test-set performance was lower than in the earlier optimized VGG16 study, illustrating sensitivity to architecture, training strategy, and dataset characteristics	Partial; validation checkpointing, no explicit curves identified
Urooj et al. 2024 [64]	BAC segmentation and quantification; patch-based multitask U-Net with auxiliary patch-position prediction	Internal: 398 screening mammograms, divided into 512 × 512 patches; external: 7264 women from Mayo Clinic BioBank at 3 sites	Internal heavy-BAC annotations by 3 expert radiologists; spline annotations converted to 16-pixel-wide segmentation masks; external Mayo data had no pixel-level BAC annotations	Internal 80%/10%/10% image split: 318/40/40 images yielding 9643/1218/1241 patches; image-level separation prevented leakage	Yes—7264-subject Mayo Clinic BioBank cohort, albeit without pixel-level segmentation ground truth	Internal segmentation: recall 61.23%; F1/Dice 57.39%; Jaccard 42.53%; precision 57.77%. External evaluation focused on BAC-score associations with cardiovascular outcomes rather than pixel-level segmentation accuracy	Provides large external clinical evaluation, but external segmentation accuracy could not be quantified because Mayo images lacked BAC outline annotations	Not reported; two-stage training and ablation analyses
Mobini et al. 2023 [17]	Binary BAC classification and weakly supervised localization/quantification; VGG16-based CNN + Grad-CAM++	1493 women; 5972 FFDM views; bilateral CC/MLO; Giotto IMAGE 3D/TOMO systems (IMS)	Image-level BAC labels from expert readers; manual BAC length served as reference for quantitative localization analysis	Patient-level split; after undersampling: 1640 training images, 888 validation images, 916 independent test images	No—single-center, single-manufacturer dataset	Classification: test AUC-ROC 0.94–0.95; F1 0.76–0.80. Quantification/localization: Spearman ρ = 0.88 with manually measured BAC length in 112 MLO views	Strong image-level discrimination without pixel-level training annotations; quantitative localization analysis was limited to 112 views, and external validation was not performed	Reported; training-loss curves
Guo et al. 2021 [16]	BAC segmentation and quantitative burden assessment; SCU-Net	Cohort A: 661 FFDM images from 216 subjects for model development; Cohort B: 10 subjects for breast-CT comparison; Cohort C: 26 subjects/961 images for longitudinal analysis; Hologic scanners	Manual whole-image segmentation by 4 annotators, including an experienced physician; breast CT calcification volume/mass used as an additional quantitative reference in 10 subjects	Cohort A randomly divided into 527 training and 134 validation images; 3455/901 effective training/validation patches; no independent test set reported	No—single institution and single scanner manufacturer	Segmentation: whole-image Dice 0.729; Jaccard 0.581; recall 0.789. Quantification: R^2^ > 0.95 vs. manual masks; R^2^ up to 0.843 for CT calcification volume and 0.873 for CT calcium mass	Detailed segmentation and quantitative validation, but single-vendor development and absence of independent external testing limit transportability	Not reported
AlGhamdi et al. 2020 [58]	Pixel-level BAC segmentation/detection; DU-Net with dense U-Net connectivity	826 FFDM images from CBIS-DDSM: 413 BAC-positive and 413 BAC-negative mammograms; CC/MLO views analyzed separately	BAC boundaries manually drawn by 3 radiologists; final annotations reviewed/corrected by the most experienced reader and used as ground truth	5-fold cross-validation on the 826-image dataset	No independent external validation	Segmentation: F1/Dice 0.922; Jaccard 0.852; sensitivity 91.2%; specificity 92.0%; accuracy 91.5%	Strong internal cross-validation performance, but the model was developed and tested on the same curated public dataset; cross-institutional and multivendor transportability was not evaluated	Not reported
Wang et al. 2017 [57]	Pixel-wise patch-based BAC detection and calcium-mass quantification; 12-layer CNN	840 FFDM images from 210 cases; 4 standard CC/MLO views per case; 23 machines across 9 facilities; GE Senographe Essential and Hologic Lorad Selenia systems	Two-round expert reader study; BAC boundaries reviewed by paired readers and consensus/corrected annotations used as ground truth	Case-based 3-fold cross-validation; within each run, remaining data further divided into training and validation sets; all 4 views from a case kept in the same subset	No independent external validation, although acquisition was multisite and multivendor	Detection: at 60% TP rate, 0.4762 cm^2^ mean FP area. Quantification: BAC area Pearson r = 0.9386; calcium-mass R^2^ = 0.9624 vs. reader-derived ground truth	Foundational study with multisite/multivendor acquisition and rigorous reader annotation, but evaluation relied on internal cross-validation rather than an independent external cohort	Not reported

Abbreviations: AI, artificial intelligence; AUC, area under the receiver operating characteristic curve; BAC, breast arterial calcification; CC, craniocaudal; CNN, convolutional neural network; CT, computed tomography; DBT, digital breast tomosynthesis; DM, digital mammography; FFDM, full-field digital mammography; FP, false positive; MLO, mediolateral oblique; TP, true positive.

**Table 2:** Principal studies of artificial intelligence-based assessment of breast arterial calcification.

Study	Design, Population, and Follow-Up	AI-Derived BAC Measure	Unit of Analysis	Cardiovascular Outcome/Reference	Validation/Risk Adjustment	Primary Prognostic Metric(s)	Critical Interpretation
Dapamede et al. 2026 [20]	Retrospective cohort from 2 healthcare systems; 123,762 women	Automated BAC area categorized as absent, mild (>0–10 mm^2^), moderate (>10–25 mm^2^), or severe (>25 mm^2^)	Patient level	MACEs	Validation across 2 healthcare systems; associations evaluated after adjustment for PREVENT risk estimates	Compared with absent BAC, PREVENT-adjusted MACE risk was approximately 30% higher with mild BAC, 75% higher with moderate BAC, and 2.8- to 3.3-fold higher with severe BAC across cohorts	Largest validation cohort; consistent dose–response association across health systems, but retrospective design and no evidence that BAC-guided management improves cardiovascular outcomes
Nerlekar et al. 2026 [67]	Multicenter retrospective cohort; 21,514 women without known CVD; mean follow-up 4.7 years; 828 MACEs	Age-adjusted BAC burden/percentile, interpreted relative to age-specific distribution	Patient level	MACEs	Evaluated across conventional ASCVD risk categories with adjustment for cardiovascular risk	Among women classified as low ASCVD risk, BAC below and above the age-specific median was associated with adjusted HRs of 1.66 and 2.31, respectively	Suggests that residual cardiovascular risk may be identified among conventionally low-risk women; clinically actionable BAC thresholds and management implications remain untested
Ibrahim et al. 2026 [65]	Retrospective case–control analysis; 3190 women with MACEs and 6458 controls	Binary, area-based, intensity-based, and combined automated BAC grading; area-based categories included mild, moderate, and severe BAC	Patient level	MACEs	Multivariable prognostic modeling with conventional cardiovascular risk factors; incremental discrimination evaluated by adding BAC to the clinical model	Area-based BAC: adjusted HRs of 1.13, 1.30, and 1.58 for mild, moderate, and severe BAC, respectively; addition of BAC changed 5-year AUC from 0.645 to 0.646	Demonstrated an independent graded association with MACEs but virtually no improvement in model discrimination, illustrating that prognostic association does not necessarily imply incremental predictive utility
Allen et al. 2024 [19]	Retrospective cohort; 18,092 women; median follow-up 6 years	Automated quantitative BAC score evaluated according to BAC presence and quartiles of burden	Patient level	Mortality and composite cardiovascular outcome comprising MI, heart failure, stroke, or death	Multivariable adjustment; dose–response assessed across BAC quartiles	Mortality: 7.8% with BAC vs. 2.3% without BAC; composite outcome: 12.4% vs. 4.3%; adjusted risk increased progressively across BAC quartiles	Large longitudinal cohort demonstrating a dose–response relationship; however, the study did not establish whether incorporating BAC into clinical management improves outcomes
Rose et al. 2024 [55]	Retrospective selected cohort; 99 women, including 26 with MACEs	Continuous proprietary AI-derived BAC score	Patient level	MACEs	Association evaluated after adjustment for age and ASCVD risk; no time-to-event analysis	Mean BAC score 35.3 with MACEs vs. 15.1 without MACEs (*p* = 0.01); adjusted OR per BAC-score unit 1.02 (95% CI, 1.00–1.04)	Exploratory evidence; small selected sample, high event prevalence, and absence of time-to-event analysis limit precision and generalizability

Abbreviations: AI, artificial intelligence; ASCVD, atherosclerotic cardiovascular disease; AUC, area under the receiver operating characteristic curve; BAC, breast arterial calcification; CI, confidence interval; CVD, cardiovascular disease; HR, hazard ratio; MACE, major adverse cardiovascular event(s); MI, myocardial infarction; OR, odds ratio; PREVENT, Predicting Risk of Cardiovascular Disease EVENT.

## Data Availability

No new data were created or analyzed in this study. Data sharing is not applicable to this article.
